# Analytical validation of a novel point‐of‐care immunoassay for canine N‐terminal pro‐brain natriuretic peptide analysis

**DOI:** 10.1111/vcp.13101

**Published:** 2022-03-20

**Authors:** Kendal E. Harr, Sonya G. Gordon, Ryan D. Baumwart, Ross Feldgreber, Matthew R. Spiro

**Affiliations:** ^1^ URIKA, LLC Mukilteo WA USA; ^2^ Department of Small Animal Clinical Science, College of Veterinary Medicine and Biomedical Science Texas A&M University College Station TX USA; ^3^ Department of Veterinary Clinical Sciences, College of Veterinary Medicine Washington State University Pullman WA USA; ^4^ Blue Pearl Veterinary Partners Northfield IL USA; ^5^ Cottage Veterinary Hospital Parker CO USA

**Keywords:** biomarker, dog, ELISA, heart, overload, stretch

## Abstract

**Background:**

N‐terminal pro‐brain natriuretic peptide (NT‐proBNP) is a widely used point‐of‐care (POC) cardiac biomarker in human medicine. Canine NT‐proBNP is used less in veterinary medicine, possibly due to the lack of a POC canine NT‐proBNP assay resulting in temporal delay, increased degradation in transport, and high reported variability in the available assay. A new quantitative POC analyzer allows fast, onsite measurement of NT‐proBNP, minimizing preanalytical error and reducing variability.

**Objective:**

We aimed to analytically validate an NT‐proBNP assay (Vcheck) according to American Society of Veterinary Clinical Pathology (ASVCP) and Clinical Laboratory Improvement Amendments (CLIA) specifications.

**Methods:**

Archived and prospective plasma and serum samples were collected from male and female, client‐owned dogs of various breeds with cardiac abnormalities (*n* = 81) and a healthy control population (*n* = 225). Precision, accuracy, analytical sensitivity, and specificity, and other statistical analyses were performed.

**Results:**

Imprecision was considered acceptable with a coefficient of variation ranging from 9% at 4000 pmol/L to 20% at 600 pmol/L. The lower limit of quantitation was 650 pmol/L based on repetitive measures evaluation. Comparison of the Vcheck assay with the Cardiopet NT‐proBNP assay revealed an excellent correlation with minimal bias when preanalytical factors were controlled. Significant degradation of NT‐proBNP occurred when current methods were used at refrigerated and room temperatures, which could change diagnostic and prognostic decision‐making. Age‐partitioned reference intervals have high reference values of 750 pmol/L and 1280 pmol/L for juvenile and adult dogs, respectively.

**Conclusions:**

The Vcheck NT‐ProBNP assay provides analytically acceptable results. Onsite testing can minimize variability related to preanalytical error and provide clinically useful contemporaneous results. Samples should be centrifuged immediately and analyzed within 2 hours of collection.

## 1 INTRODUCTION

**FIGURE 1:**
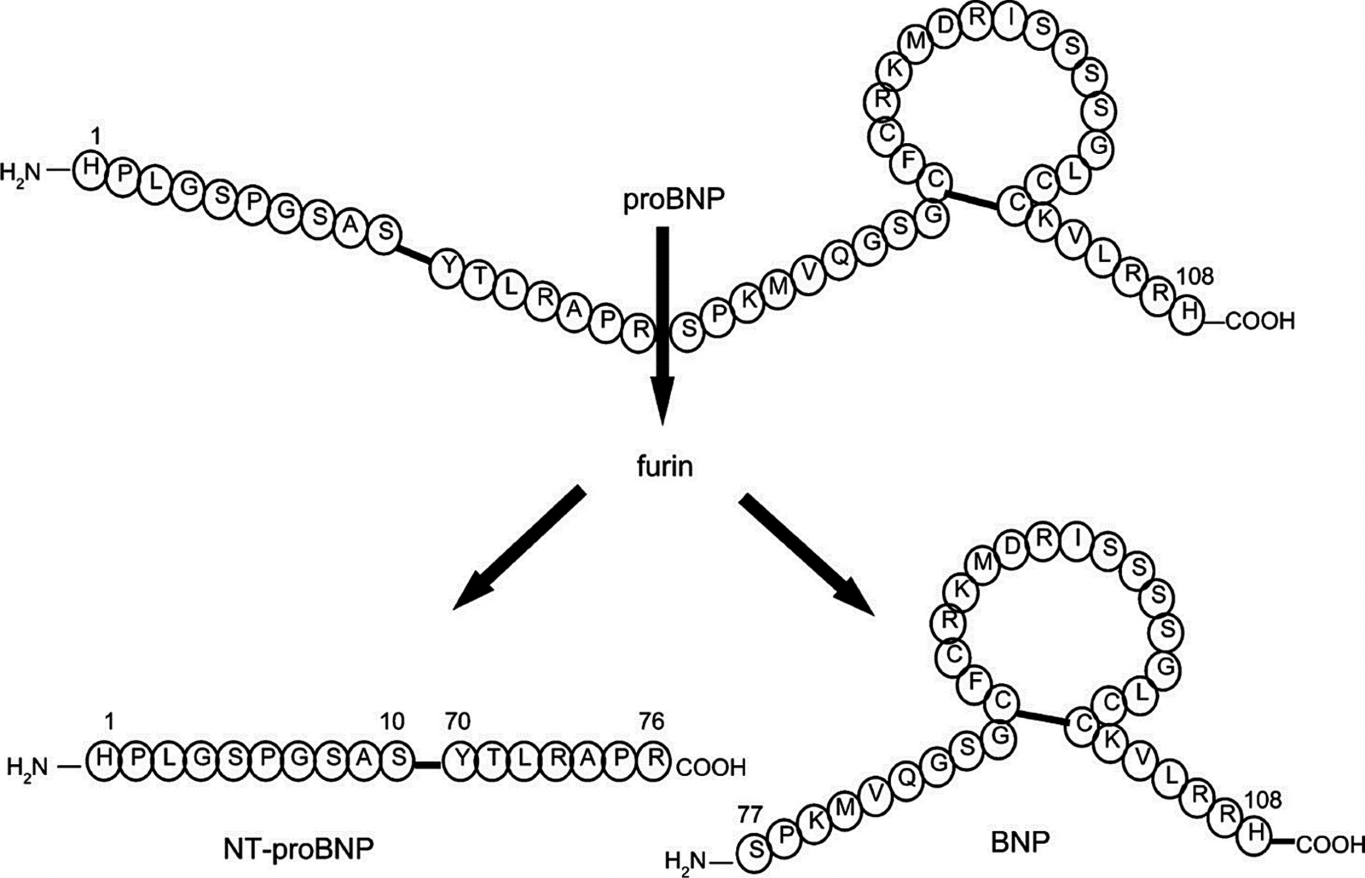
Production of NT‐proBNP and BNP. proBNP is cleaved by furin to produce the NT‐proBNP and BNP molecules when these compounds are released in response to myocardial stretch. From Hall C. Essential biochemistry and physiology of (NT‐pro)BNP. Eur J Heart Fail. 2004;6:257‐260. doi:10.1016/j.ejheart.2003.12.015. Used with permission. NT‐proBNP, N‐terminal pro‐brain natriuretic peptide

B‐type natriuretic peptide (BNP) is a 32‐amino acid cardiac natriuretic peptide hormone that is secreted into the circulation by cardiac myocytes and fibroblasts in response to myocardial stress or stretch of the heart's walls due to increased volume and pressure, and its production is therefore significantly upregulated in cardiac failure. BNP binds to receptors in organs such as the kidney, stimulates increased intracellular cGMP production, and induces diuresis, vasodilation, inhibits renin and aldosterone production, and inhibits cardiac and vascular myocyte growth, and possibly inhibits fibroblast proliferation. BNP is secreted as a prohormone, proBNP, and then cleaved into the biologically active hormone, BNP, and the nonactive amino‐terminus, NT‐proBNP (76 amino acids); therefore, the concentration of either can be used to assess the magnitude of myocardial wall stress or stretch (Figure 1).^1^ However, NT‐proBNP is more stable and has a longer half‐life than both the prohormone and BNP, making it a useable diagnostic analyte. When antibodies are targeted to the C‐terminal end or central regions of NT‐proBNP, measured concentrations of NT‐proBNP are less susceptible to degradation.^2^ For the past two decades, NT‐proBNP and other natriuretic peptides have been explored as valuable biomarkers for cardiac disease in both veterinary and human medicine.

In people, NT‐proBNP has been thoroughly analytically and diagnostically validated. NT‐proBNP is relatively inexpensive, easily and rapidly measured, reflects a pivotal pathophysiologic pathway, provides additional novel information to available clinical data, and has been proven to facilitate the diagnosis, prognosis, and management of human heart failure, thereby approximating an ideal cardiac biomarker.^3^ NT‐proBNP is currently used as a prognostic indicator in human patients and appears to predict adverse outcomes better than other cardiac biomarkers upon patient follow‐up, suggesting the ability to aid in risk stratification.^4^ Additionally, a metanalysis of 18 research studies revealed that high NT‐proBNP concentrations, indicating cardiac stretch, were associated with a higher risk of death due to acute respiratory distress syndrome for up to 60 days.^5^ Thus, NT‐proBNP is an important prognosticator for both left heart and right heart overload, which includes either cardiogenic or pulmonogenic etiologies.

In dogs, analytical and diagnostic validation has been investigated for an NT‐proBNP ELISA assay^6^ (IDEXX, Westbrook, ME, USA).^7^, ^8^ There are significant reported differences between human and dog NT‐proBNP, including reference values, diagnostic thresholds, and stability. Previously reported canine reference intervals have ranged up to 900 pmol/L in the general adult dog population,^9^ with some breed variability in optimized decision thresholds; such as a cutoff value of 457 pmol/L in Doberman pinschers has been reported to accurately identify occult dilated cardiomyopathy, when used in combination with a Holter monitor.^10^, ^11^ In contrast, in greyhounds, which also have relatively high cardiac troponin concentrations, the mean NT‐proBNP concentration in a population of healthy greyhounds was found to be 946 pmol/L.^12^ In comparison, human reference values tiered by age and sex can be <20 pmol/L at younger ages. (https://www.mayocliniclabs.com/test‐catalog/Clinical+and+Interpretive/84291) Equally important is a documented marked difference in compound stability at all temperatures, which varies in the human and veterinary literature.^6^, ^13^

The very short half‐life of canine BNP (as little as 90 seconds), which enables quickly tailored physiologic adjustments in the body, makes the active hormone unusable as a cardiac biomarker in the dog. Cahill et al reported stability of canine NT‐proBNP at room temperature (25°C) as 90% and 76% retention of baseline concentrations at 48 hours in plasma and serum samples, respectively.^6^ However, Collins et al reported stability at room temperature (25°C) as 29% retention of baseline concentrations at 48 hours in serum samples, consistent with other studies.^13^, ^14^, ^15^ In contrast, in people, NT‐proBNP is reported to have variable stability at room temperature (25°C), retaining 92% to 10% of baseline concentrations at 72 hours in plasma and serum, respectively.^13^ The discrepancies in preanalytical degradation in canine NT‐proBNP could impact the research and clinical interpretation of this assay. Indeed, Collins et al reported that approximately 80% of canine serum samples had clinically relevant differences in NT‐proBNP concentrations, resulting in a different diagnosis after samples were stored for 48 hours at room temperature.

This study sought to fully characterize analytical performance, including preanalytical and analytical factors, and verify manufacturer's claims for canine NT‐proBNP (Bionote, Hwaseong‐si, Gyeonggi‐do, South Korea) measured using V200 and V2400 Vcheck analyzers (Bionote). This is a novel in‐clinic analyzer that minimizes time to analysis so that a more robust diagnostic performance assessment can be completed. Validation was carried out according to American Society of Veterinary Clinical Pathology (ASVCP) guidelines and Clinical Laboratory Improvement Amendments (CLIA) specifications and included a bias comparison with the marketplace assay (Cardiopet NT‐proBNP ELISA; IDEXX Bioanalytics, West Sacramento, CA, USA), precision analyses, analytical sensitivity verification of the limit of quantitation (LOQ), analytical specificity verification of interferent effects, and generation of reference intervals in a normal population of dogs.

## 2 MATERIALS AND METHODS

### 2.1 Animal recruitment

This analytical verification study included prospectively collecting (*n* = 61) samples from male and female, adult client‐owned dogs of various breeds with possible cardiac abnormalities, paired samples archived from a previous study using cavalier King Charles spaniels in an ultracold freezer (−80°C) (*n* = 20), and samples from a healthy control population (*n* = 225).

To evaluate preanalytical error and the two methods, dogs with possible cardiac abnormalities (*n* = 61 total; *n* = 56 with cardiac abnormality, *n* = 5 normal) were prospectively recruited from dogs presented for cardiac evaluation at the Texas A&M University Veterinary Teaching Hospital, College Station Texas (IACUC# 2020‐0193C), Washington State University, Pullman, WA, USA (IACUC# ASAF#6858), and the Blue Pearl Veterinary Referral Center, Northfield, IL, USA between October 2020 and March 2021. Informed consent was obtained from all owners. The diagnostic evaluation for dogs with cardiac abnormalities included a history, complete physical examination, creatinine concentrations, and routine echocardiographic examination performed by either a board‐certified cardiologist or a cardiology resident under the direct supervision of a board‐certified cardiologist. If respiratory symptoms were reported by the owners or noticed during physical examination, chest radiographs were taken.

Dogs included in the healthy control reference interval population (*n* = 225) were recruited from a population of healthy male and female, juvenile to adult dogs presented for preanesthetic screening for either surgery or dentistry at a general practice clinic. The health of the reference population was established based on history, physical examination, laboratory diagnostics, including creatinine, an uneventful anesthetic event, and no clinical signs or medication (aside from preventive therapies) 4‐8 months postsampling. Echocardiography was only performed if indicated by an increased NT‐proBNP concentration (>1000 pmol/L) as no clinical signs were present in the apparently healthy population. Exclusion criteria included the presence of a murmur, an abnormal echocardiogram result, physical examination not supportive of health, renal, or other disease evidenced on blood work, such as a creatinine concentration moderately outside of reference intervals, lack of requested follow‐up visit or information, and mortality or prescribed cardiac medication within 8 months postprocedure.

All data regarding history, physical examination, and diagnostic findings were recorded in a standardized data sheet.

### 2.2 Sample handling, collection, and transport

Sample handling is summarized for the different parts of the verification as follows:

1. Precision: Twenty replicate analyses were performed for four canine serum pools at calculated target concentrations (350, 600, 1900, and 4000 pmol/L) over the course of approximately 5 hours, required by a single Vcheck instrument. Samples were maintained in the refrigerator (4°C) over this time period and warmed to room temperature just prior to analysis.
2. Accuracy: This was assessed using archived samples (*n* = 20) collected on January 1, 2020, as part of a separate clinical study using cavalier King Charles spaniels diagnosed with myxomatous mitral valve disease. The archived samples (*n* = 20) were analyzed within 10 months of storage in an ultracold (−80°C) freezer. Ethylenediaminetetraacetic acid (EDTA) plasma and serum aliquots were retrieved from an ultracold freezer (−80°C) and shipped on dry ice, expedited overnight, for off‐site analysis of NT‐proBNP by the Vcheck analyzer (serum) at the URIKA (URIKA, LLC, Mukilteo, WA, USA) and a Cardiopet NT‐proBNP ELISA (plasma) (IDEXX Bioanalytics). Preanalytical conditions, including one freeze‐thaw cycle for all samples, were kept as similar as possible for these paired samples.
3. Sample degradation: Ten serum samples were measured once, refrigerated (4°C) for approximately 14 hours, and then remeasured to assess potential degradation in serum samples overnight as measured by the Vcheck assay. The same experiment was repeated at room temperature (20°C).
4. Preanalytical error: In a prospective comparison designed to replicate the real‐world sample submission process, paired serum and plasma samples were analyzed using the Vcheck NT‐proBNP assay in‐clinic and transported according to the standard clinical submission protocol of the facility to the regional IDEXX reference laboratory for analysis using the CardioPet NT‐proBNP assay. For prospective fresh samples from the referral centers, standard venipuncture was performed, plasma samples were collected in K3‐EDTA tubes, and serum samples were collected into tubes lacking anticoagulant that did not have a serum separator. Centrifugation of both sample types was performed within 1 hour of collection. Serum samples for the Vcheck analysis were analyzed within 2 hours of sample collection at all facilities. Fresh, refrigerated plasma samples for Cardiopet analysis were shipped by IDEXX courier or Fed Ex on wet ice as per the manufacturer's instructions, typically within 24 hours of sample collection from the Washington and Illinois facilities; rare plasma samples collected in Washington were stored from 4 to 7 days in a refrigerator, prior to pick up by the IDEXX courier. As per the facility's protocol, plasma samples from Texas A&M were frozen overnight after centrifugation and transported on cold packs in a cooler to the reference laboratory by IDEXX courier the following morning or the next workday. Courier and laboratory handling of the samples is unknown.

### 2.3 Measurement of NT‐proBNP

The Vcheck canine NT‐proBNP Test kit (Bionote) uses anti‐canine NT‐proBNP antibodies with conjugated fluorescence microparticles which are released, creating a signal when the antibody binds canine NT‐proBNP. The Vcheck 200 and Vcheck 2400 instruments measure final fluorescence in an identical manner, although the 200 is designed for a single dry chemical cartridge and the 2400 has a multi‐assay capacity of 24 dry chemical cartridges at one time. All instruments were calibrated using Bionote's recommended software and specifications prior to analysis. Currently, there are no available assayed quality control materials (QCMs) with a concentration in the linear range of the canine assays. The assay was performed in accordance with the manufacturer's instructions.^16^ Briefly, patient‐side, 100 μL of serum was placed in the supplied diluent tube and mixed by pipetting. The mixture was loaded to the well present in the dry chemical cartridge, which was inserted in the instrument. Results are reported by the instrument after a 15‐minute incubation. The LOQ of 500 pmol/L has been set by the manufacturer, and any concentration less than that is reported as <500 pmol/L.

The Cardiopet NT‐proBNP (IDEXX) is described as a second‐generation canine NT‐proBNP sandwich ELISA. It includes an increased number of calibrators with increasing concentrations of NT‐proBNP peptide in a protein buffer base as well as three controls (low, medium, and high) that are routinely analyzed and provide internal quality control for each assay run.^6^ Ethylenediaminetetraacetic acid plasma was recommended as the best sample for Cardiopet NT‐proBNP at this time upon phone consultation with IDEXX representatives; although online sampling directions state that serum samples are acceptable, but must be refrigerated. This was presumed to be due to differences in reported instability of plasma vs serum at refrigerated and room temperatures.^6^ Therefore, this study submitted plasma samples based on verbal recommendations by the manufacturer that this was a preferred sample type. All plasma samples delivered to IDEXX were transported within the current manufacturer recommendations: “Storage and Stability: 7 days at 2‐8°C; freeze for longer storage.”^17^ No LOQ has been set by the manufacturer, and all quantitative values are presented as per the manufacturer's recommendations.^17^

### 2.4 Statistical analysis

All statistical analyses were performed using MedCalc version 20 to 64‐bit software (MedCalc software Ltd, Ostend, Belgium). The coefficient of variation (CV) was calculated using 20 replicates at target concentrations (350, 600, 1900, and 4000 pmol/L), spanning approximately half of the assay's published linear range (500‐10 000 pmol/L) and three times the upper reference limit. Methods were compared using linear regression and Bland‐Altman plots, with calculated bias and other descriptive statistics.^18^ Total analytical error was calculated using modified (2 × CV) + bias.^19^ The lower LOQ was verified and modified using an acceptable CV (<20%) in canine serum samples using a repetitive measures study of the target matrix. Reference intervals were calculated according to the guidelines from the ASVCP, using Tukey's outlier analysis with a *z* < 0.05, the Robust method for <120 reference values, and the recommended nonparametric analysis for datasets with >120 reference values.^20^ To assess clinical differences, the number of values with a different classification or diagnosis was divided by the total number of values and multiplied by 100.

## 3 RESULTS

### 3.1 Precision and LOQ

Imprecision varied across concentrations with the lowest CV at the highest concentration as expected (Table 1). The precision of analysis below the LOQ was investigated with a 20× replicate study of pooled canine serum samples with a target concentration of <500 pmol/L as measured by the Vcheck analyzer and approximately 350 pmol/L as measured by the Cardiopet assay. Of 20 replicates of one low concentration sample, 15 values of <500 pmol/L were correctly reported, with the five remaining results ranging between 522 and 611 pmol/L.

**TABLE 1:** Precision of the Vcheck NT‐proBNP assay across concentrations

Target concentration(pmol/L)	Coefficient of variation(%)
600	20
1900	12
4000	9

Abbreviation: NT‐proBNP, N‐terminal pro‐brain natriuretic peptide.

Based on imprecision studies in which the CV reaches 20% at a target concentration of 600 pmol/L and the presence of spurious values up to 611 pmol/L in sample results with a target concentration of <500 pmol/L, the recommended LOQ is increased to 650 pmol/L.

### 3.2 Accuracy

#### 3.2.1 Bias—Instrument to instrument comparison

**FIGURE 2:**
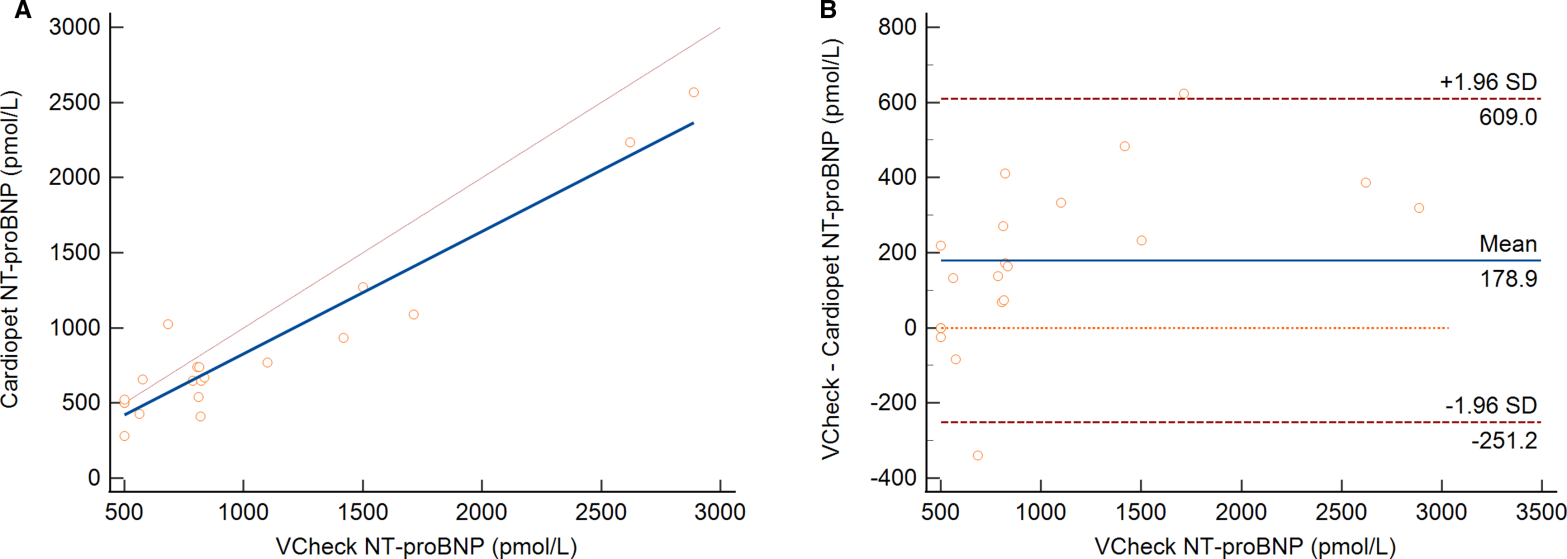
Comparison of the Vcheck and Cardiopet NT‐proBNP assays using (A) linear regression (*y* = *mx* + *b*) and (B) Bland‐Altman plots. Direct instrument‐to‐instrument comparisons of *n* = 20 frozen paired serum and EDTA plasma samples from the same dogs at the same time were performed. All samples were handled identically and shipped on dry ice. Excellent correlations were revealed between the marketplace and Vcheck analysis. The line of equality, *x* = *y* (fine brown line), and the regression line (blue) are presented. EDTA, ethylenediaminetetraacetic acid; NT‐proBNP, N‐terminal pro‐brain natriuretic peptide

Twenty paired archival frozen samples (one freeze‐thaw cycle) were used in an instrument comparison of the Vcheck serum analysis performed at reference laboratory (URIKA, LLC) to the Cardiopet NT‐proBNP ELISA plasma analysis (IDEXX Bioanalytics) and showed excellent correlation. The linear equation was *y* = 0.9*x* + 37 with 95% confidence intervals (CIs) and a slope of 0.75‐1.05 and intercept of −150 to 224 (Figure 2A). The coefficient of determination (*R* ^2^) was 0.9. The mean difference found using the Bland‐Altman analysis was 179 pmol/L (Figure 2B). The mean (median) and range for these 20 samples were 944 (746), 293‐2904 pmol/L for the Cardiopet ELISA, and 1013 (812), <500‐2888 pmol/L for the Vcheck NT‐proBNP assay, respectively. Note that Bionote, the manufacturer of the VCheck, had set an LOQ of 500 pmol/L at the time of this study while there was no LOQ established for the Cardiopet ELISA. The instrument results were not significantly different using a paired *t* test.

#### 3.2.2 Bias—Prospective fresh sample comparison

**FIGURE 3:**
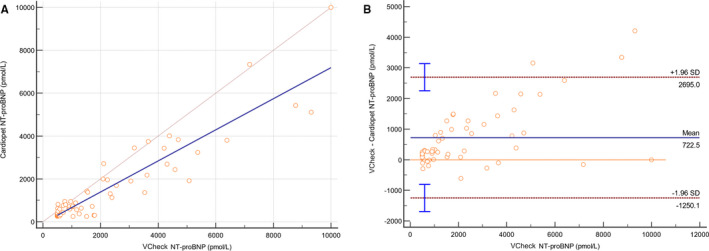
Comparison of the Vcheck and Cardiopet NT‐proBNP assays using (A) Linear regression (*y* = *mx* + *b*) and (B) Bland‐Altman plots. Real‐world comparisons contain evidence of preanalytical error. Sixty‐one fresh serum and plasma samples collected at the same time from the same dogs revealed a markedly different linear comparison. The line of equality, *x* = *y* (fine brown line), and the regression line (blue) are presented. NT‐proBNP, N‐terminal pro‐brain natriuretic peptide

When prospectively comparing fresh samples analyzed by the Vcheck point‐of‐care (POC) analysis to the Cardiopet ELISA performed at the reference laboratory, 61 paired serum and plasma samples, collected at the same time from the same dogs, generated a significantly different linear equation, *y* = 0.7*x* − 52 (Figure 3A). The slope of the line did not include 1, with a 95% CI of 0.63‐0.81. Additionally, the 95% CI of the intercept was much larger, ranging from −278 to 268. The coefficient of determination (*R* ^2^) decreased to 0.8. The mean difference found using the Bland‐Altman analysis was 723 pmol/L (Figure 3B).

### 3.3 Observed total error

Bias could not be ascertained in this assay as there is no gold standard method for canine NT‐proBNP and the concentration of commercially available QCM (constructed to assess human assays) was below the linear range of the canine assay. Therefore, total error was estimated using 2 × CV. It is expected that the total observed error in the Vcheck NT‐proBNP assay will range from approximately 40% at 600 pmol/L to 18% at 4000 pmol/L, with a further linear decrease in CV, and therefore total error, expected at higher concentrations.^6^

### 3.4 Preanalytical error

Using the Vcheck assay, an approximate 20% loss from original mean concentration was documented in a 14‐hour time period when 10 original, unaltered canine serum samples were stored at 4°C, with samples ranging from 16% to 33% loss during this time period. Ten samples were then purposefully left at room temperature (20°C) overnight. Measurement the next day revealed that all samples had lost at least 50% of their concentration, which would result in significant differences in clinical interpretation in all samples.

The mean, (median), and range for the 61 prospective samples using the Cardiopet ELISA and the Vcheck NT‐proBNP assays were 1714 (799), 250 to >10 000 pmol/L, and 2404 (1331), <500 to >10 000 pmol/L, respectively. The greatest difference in the measured concentration (Vcheck‐Cardiopet) of 4205 pmol/L (9305‐5110 pmol/L) was found at concentrations at the high end of the linear range (10 000 pmol/L). The highest percent loss was 84% (1519 – 250 ÷ 1519) and was found at concentrations at the lower end of the linear range. Using the reference intervals recommended by both manufacturers at the time of this study (<900 pmol/L), the difference in NT‐proBNP concentrations between paired samples would result in a different clinical interpretation in 15% (9/61) of the submitted samples. Given the positive bias for the Vcheck assay, use of this assay would result in more abnormal test results compared with the Cardiopet assay; that is, invariably, the Vcheck samples would indicate increased myocardial stretch with the Cardiopet assay reporting values within the reference interval. The percent difference in result interpretations varied between veterinary facilities from 0% (0/14 dogs) to 28% (6/21 dogs).

### 3.5 Reference intervals

**FIGURE 4:**
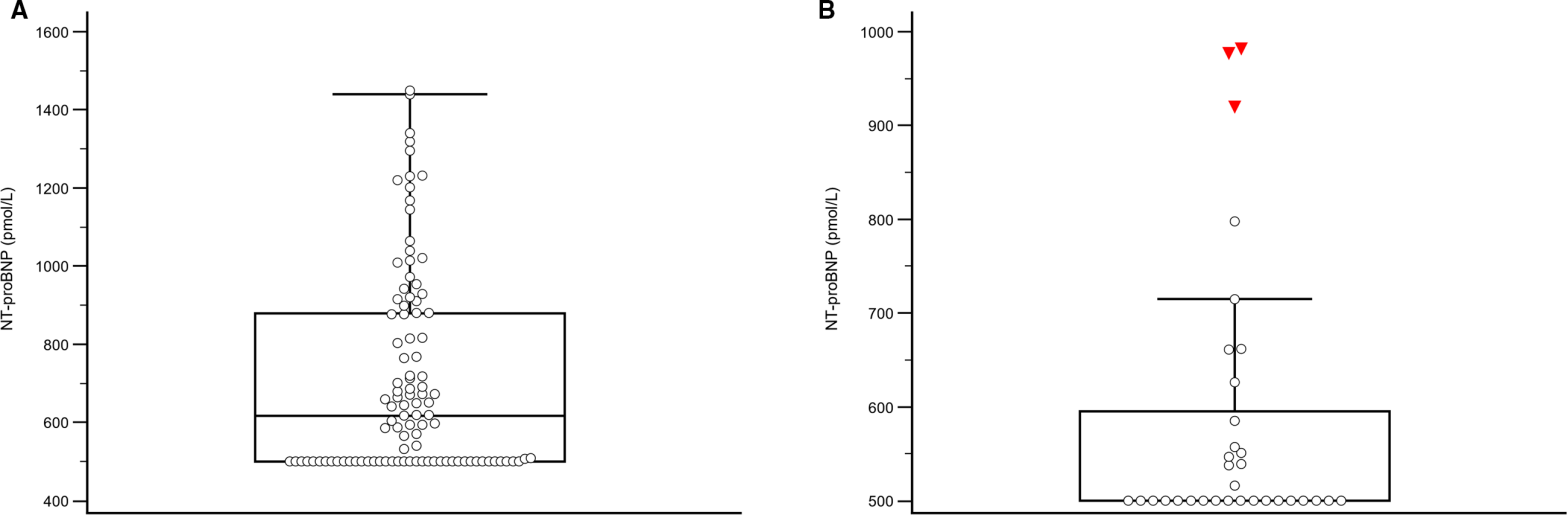
Nonparametric distributions of the serum NT‐proBNP concentrations generated using the Vcheck assay. (A) A reference population was composed of 125 apparently healthy, adult, male, and female dogs between 2 years and geriatric ages. The lower LOQ was set to 500 pmol/L during this study. The central line of the quartile box represents the median (617 pmol/L). The 95% medical reference interval is <500–1280 pmol/L after six outliers were removed using Tukey's outlier analysis. (B) A reference population was composed of 36 apparently healthy, juvenile, male, and female dogs between 0 and 18 months. The lower LOQ was set to 500 pmol/L during this study. The lower line of the quartile box represents the median (<500 pmol/L) as the majority of the results generated from the juvenile population were below the LOQ of this assay. The 95% medical reference interval is <500–750 pmol/L after three outliers were removed using Tukey's outlier analysis. LOQ, limit of quantitation; NT‐proBNP, N‐terminal pro‐brain natriuretic peptide

Apparently, healthy dogs (*n* = 225) that met inclusion and exclusion criteria were separated into three age‐partitioned categories of juvenile (0‐18 months, *n* = 36), adult (19 months to geriatric age, *n* = 125), and geriatric (*n* = 64) based on previously published weight vs age charts.^21^ The distribution of NT‐proBNP is nonparametric (*P* < 0.0001) based on a Kolmogorov‐Smirnov test (Figure 4A). From the apparently healthy adult dogs (*n* = 125) six outliers were removed using Tukey's outlier analysis resulting in 119 remaining values having a median ± standard deviation, minimum/maximum range of 617 ± 257, <500‐1450. Using the nonparametric percentile method,^20^, ^22^ the 95% medical reference interval is <500‐1280 pmol/L with a 90% CI of the upper reference limit of 1168‐1440 pmol/L. From the apparently healthy, juvenile dogs (*n* = 36), three outliers were removed using Tukey's outlier analysis resulting in 33 remaining values having a median ± standard deviation, minimum/maximum range of <500 ± 142, <500‐982 pmol/L. (Figure 4B) The 95% medical reference interval of <500‐750 pmol/L with a 90% CI of the upper reference limit of 719‐884 pmol/L was generated using the Robust method.^20^, ^22^

A reference interval was not created for the geriatric population (*n* = 64) as the authors believed that this population likely contained animals with subclinical disease based on previously known disease prevalence in this population with increased NT‐proBNP values and increased variation in NT‐proBNP results.^18^, ^21^ Diseased animals could not be reliably separated using outlier statistics in this nonparametric dataset with the available diagnostic workup. Descriptive statistics demonstrated a mean (median) ± SD, minimum/maximum interval of 1001 (945) ± 731, <500‐3783 pmol/L, respectively.

### 3.6 Interferents (analytical specificity)

Spiking 10 samples across the linear range of the assay with 35 mg/dL hemoglobin (bovine hemoglobin; EMD Millipore, Burlington, MA, USA) and 1000 mg/dL Intralipid (Baxter International, Inc., Deerfield, IL, USA) did not reveal a statistically significant difference using paired *t* tests for reported values beyond the known total error (*P* > 0.05). Table 2 shows manufacturer‐reported interference interactions.

**TABLE 2:** Analytical specificity of the Vcheck NT‐proBNP assay with known interferents

Interfering substance	Conc. (mg/dL)	Interference
Hemoglobin	≤37.5	None
Intralipid	≤1200	None
Bilirubin	≤5	None
Vitamin C	≤1000	None

Abbreviation: NT‐proBNP, N‐terminal pro‐brain natriuretic peptide.

## 4 DISCUSSION

Results of this study demonstrate that the Vcheck NT‐proBNP assay is a valid point‐of‐care cardiac biomarker using canine serum. The CLIA (the federal law governing the performance of medical diagnostic laboratories) states that precision, analytical sensitivity, accuracy, analytical specificity/interfering substances, reportable ranges, reference intervals, and “any other performance characteristic required for test performance” should be thoroughly investigated and verified for validation.^23^ In this case, preanalytical error and sample handling, specifically temperature and time from collection to analysis, are performance characteristics that must be considered due to significant degradation in a relatively short period of time using both plasma and serum samples in the two methods evaluated in this study.^14^

The Vcheck precision performance was verified, and performance was deemed acceptable for an immunoassay, which typically has higher levels of imprecision, especially at lower concentrations. Currently, the ASVCP has not assigned an allowable total error (ATE) to NT‐proBNP, nor has the College of American Pathologists. Troponin I, another cardiac biomarker measured by immunoassay, was evaluated, and the ASVCP Quality and Laboratory Assurance Committee assigned 70% ATE with a maximal CV of 20%.^19^ This assay does fall within the guidelines for troponin, although this is not a tailored, optimal quality goal. Further work is needed to establish ATE for NT‐proBNP to ensure that diagnoses and clinical decision‐making is not affected by analytical error.

Based on imprecision studies in which the CV reaches 20% at a target concentration of 600 pmol/L and the presence of spurious values up to 611 pmol/L in sample results with a target concentration of <500 pmol/L, the recommended LOQ is increased to 650 pmol/L. Hence, the recommended linear range is from <650 to 10 000 pmol/L.^6^ This change does not affect clinical interpretation as reference intervals are higher than this cutoff. As no commercially available, stabilized QCM is available for the assessment of canine NT‐proBNP, we recommend that users of this analysis archive frozen aliquots with high‐ and low‐level serum samples such that only one freeze‐thaw cycle occurs. These aliquots can be used monthly or periodically to assess analytic precision, especially at low concentrations, to ensure adequate performance.

Accuracy assessment was challenging due to a lack of assayed, commercially available QCM at the needed concentration range found in dogs. Human reference intervals are also age based with high reference limits around 20 pmol/L, which is more than an order of magnitude lower than concentrations found in healthy dogs, and of course, NT‐proBNP concentrations in diseased dogs are higher. The manufacturer states that they are currently discussing the construction of a dog‐specific QCM with a QCM manufacturer, which is needed to adequately assess this assay for day‐to‐day quality in a diagnostic setting. When a set of 20 paired, archived samples with varying concentrations from <500 to approximately 3000 pmol/L was managed with optimal transport conditions, including overnight shipping and dry ice, an excellent correlation (*R* ^2^ = 0.9) with the existing marketplace assay was found with a minimal bias of 179 pmol/L. However, when fresh samples were analyzed in an in‐clinic setting (Vcheck) or shipped with commercial couriers (Cardiopet) and analyzed as recommended by the manufacturer, the *R* ^2^ fell to 0.8, and the positive bias increased to >700 pmol/L. This positive bias of the on‐site Vcheck assay compared with samples shipped to the regional reference laboratory is consistent with sample degradation, as has been previously reported.^13^ When paired sample results are compared, these discrepancies are clinically relevant, affecting interpretation in approximately 15% of the samples (9/61 dogs), with a resultant difference in the designation of normal vs abnormal.

The severity of degradation (decreased measured concentrations over time) varied with the facility, which could have been due to preanalytical or analytical factors as these samples were handled and shipped differently and would likely have been analyzed at reference laboratories in different locations. Of note is the fact that the facility that had a 0% difference in clinical interpretation (0/14 dogs) was the same facility that froze plasma samples overnight prior to shipment by the courier that picked up samples inside the building. The facility with the highest difference in clinical interpretation of 28% (6/21 dogs) also had the highest total difference in concentration of the paired samples (4205 pmol/L). This facility had daily, 7 days per week, sample transport by an IDEXX courier, and samples were refrigerated prior to placement in the IDEXX outdoor sample box at a designated time. Conditions of transport and analysis after placement in the outdoor sample box are unknown. This study was designed to determine how these instruments perform in the hands of the end‐user, the veterinary practitioner and/or technician. Due to this study design, a limitation is that the samples and instruments were handled by multiple users without the direct oversight of the authors at all steps of sample handling and analysis. However, this is also a strength of this study since it recreates the real‐world scenario in which instruments are used by the intended user mimicking clinical sample analysis and submission on a day‐to‐day basis.

Significant degradation that occurred in refrigerated samples measured the next day could change diagnoses and prognoses. This is consistent with what has been previously found in dogs.^13^ Degradation increases with increased temperatures.^6^, ^13^ This means that over the course of an afternoon if left at room temperature, a sample could have a measured concentration of 25% of the original concentration. While the exact decrease in measured concentrations could change based on the targeted epitope in the assay, and the amount of degradation varies individually from sample to sample, a significantly decreased measured concentration is expected within 24 hours in samples at both refrigerated and room temperature based on previous studies in dogs.^13^ This represents a species and methodologic difference, in that human NT‐proBNP has been reported to have a half‐life of days using optimized assays. Our findings support increased variability in both fresh plasma and serum samples submitted according to the manufacturer's directions, resulting in a site‐dependent, significantly increased discrepancy of normal and abnormal NT‐proBNP values. This supports significant preanalytical variability induced by unknown temperatures during transport as well as extended times from sampling to analysis. For this reason, we recommend that serum should be separated as soon as the clot has formed, refrigerated if not analyzed immediately, and analyzed within 2 hours of collection. Serum samples must be frozen if the assay is to be performed more than 3 hours after sampling. This study did not address storage in a standard, frosted freezer (−20°C), which could be important information for facilities establishing a custom QCM for the assay or for some research archives. The manufacturer reports minimal changes in samples frozen in a standard, frosted freezer (−20°C) over a 5‐day time period with a single freeze‐thaw cycle. However, this was not verified by the authors. Therefore, further studies examining the stability of NT‐proBNP after freezing are warranted, and researchers and quality assurance specialists using archived samples are urged to assess storage changes at −20°C prior to use.

In human serum, there have been conflicting results that have been attributed to antibodies specific to different epitopes or regions of the NT‐proBNP molecule. In general, assays with antibodies targeted at terminal epitopes, especially the N‐terminal epitope, in the 76‐amino acid molecule will degrade faster as these are cleaved first during enzymatic and proteolytic degradation.^2^ It has, therefore, been recommended that antibodies be directed against epitopes in the mid‐region of NT‐proBNP, which results in more stable measured concentrations over time.^2^ Fully automated, optimized human assays might have multiple antibodies targeted to different epitopes to improve measurement stability as the molecule degrades.^24^ According to manufacturer documents, the Vcheck targets a variety of epitopes on canine NT‐proBNP.

Ethylenediaminetetraacetic acid has been reported to decrease the activity of proteases that break down the NT‐proBNP molecule in serum and has, therefore, been shown to be a more stable sample over 72 hours. Interestingly, Cahill et al reported only 4% and 19% loss from the baseline NT‐proBNP concentration in canine EDTA plasma samples at room temperature (25°C).^6^ However, in this study, we documented a significant breakdown over time in EDTA plasma samples handled with the current manufacturer's recommendations. This indicates that these recommendations should be tightened and turnaround time shortened. Cahill et al specifically note that “sample integrity studies were conducted under controlled environmental conditions. Data has not been generated to evaluate NT‐proBNP integrity at temperature >25°C to which samples may be exposed during ambient shipping in warmer climate.” Additionally, the recommended transport time in this investigative paper of 48‐hour transit times is significantly shorter than current IDEXX specimen recommendations of 7 days if refrigerated.^6^

The magnitude of the possible impact of the preanalytical factors on the reported biological variability^25^ and the diagnostic performance of canine NT‐proBNP concentrations measured by the Cardiopet assay is unknown, but any off‐site assay is likely to suffer more from these factors than a POC assay. In addition, the temporal delay in test results can be likewise overcome by a fast point‐of‐care assay. It should also be noted that sample freezing has been previously reported to result in an increased concentration using some methods.^13^ Taken together, the availability of a valid fast POC canine NT‐proBNP assay will likely contribute to better diagnostic results without preanalytical error, an increase in clinical use, and a flurry of new diagnostic validation studies.

The analysis of the healthy dog population revealed a significant difference between age categories based on previously published juvenile, adult, and geriatric categorization, which is consistent with that reported in human medicine.^21^ The current high reference value (900 pmol/L) used by both manufacturers to define a normal population is not partitioned to a level that is useful diagnostically. This nonpartitioned reference value will result in false negatives in younger animals and false positives in adult animals, especially in older adult animals. The finding that there were likely patients with subclinical ongoing cardiac stretch in the geriatric population is not surprising considering previous findings regarding the prevalence of disease. Physical and laboratory abnormalities are common in apparently healthy, elderly dogs, and this study supports the previously reported increased prevalence of subclinical cardiac disease in the geriatric population.^21^ To exclude animals with any concurrent disease, clinically relevant abnormal blood values, such as creatinine concentrations for renal disease, were used to identify individuals with potentially confounding disease. Chronic kidney disease is known to result in increased NT‐proBNP concentrations due to decreased excretion, and, by definition, the reference interval population must be comprised of healthy animals.^26^ In this geriatric dog population, owners occasionally declined referral to a cardiologist, making definitive inclusion or exclusion of increased NT‐proBNP concentrations difficult. Further studies of geriatric canine patients with complete cardiac workups to ascertain their health are needed to determine if NT‐proBNP values in geriatric patients are indeed different than those in adult dogs.

Veterinarians play a key role in implementing health screening and improving health care for elderly pets.^21^ Cardiac biomarkers that have age‐optimized reference intervals, as well as disease and breed targeted decision thresholds, will provide an additional effective diagnostic tool for this endeavor. The adult reference intervals generated in this study are higher than those previously reported by manufacturers, which might be due to the method used, immediate sample processing with no degradation, the reference population, or correctly applied, partitioned statistics in accordance with ASVCP guidelines.

## 5 CONCLUSIONS

This Vcheck validation study found that age‐partitioned reference intervals should be modified to upper reference limits of 750 and 1280 pmol/L for juvenile and adult, mixed‐sex dogs, respectively, which should improve diagnostic utility. A modification of the LOQ to 650 pmol/L is warranted due to increased imprecision at lower concentrations in the Vcheck analysis. All other manufacturer claims were verified. Preanalytical error is a significant factor for accurate NT‐proBNP measurements and should be minimized by controlling temperatures and minimizing time to analysis, preferably within 2 hours of blood collection.

## DISCLOSURE

This study was funded by Bionote USA, Inc., Big Lake, MN, USA.
